# Proteomic analysis of ceftazidime and meropenem-exposed *Pseudomonas aeruginosa* ATCC 9027

**DOI:** 10.1186/s12953-023-00217-6

**Published:** 2023-09-28

**Authors:** Hong Loan Ngo, Thuc Quyen Huynh, Nguyen Bao Vy Tran, Ngoc Hoa Binh Nguyen, Thi Hang Tong, Thi Truc Ly Trinh, Van Dung Nguyen, Prem Prakash Das, Teck Kwang Lim, Qingsong Lin, Thi Thu Hoai Nguyen

**Affiliations:** 1 School of Biotechnology, International University, Ho Chi Minh City, Vietnam; 2 Viet Nam National University Ho Chi Minh City, Ho Chi Minh City, Vietnam; 3 Research Center for Infectious Diseases, International University, Ho Chi Minh City, Vietnam; 4 Department of Biological Sciences, Protein and Proteomics Centre, National University of Singapore, Singapore, Singapore

**Keywords:** Antibiotic resistance, Ceftazidime, iTRAQ, Pseudomonas aeruginosa, Quantitative proteomics analysis

## Abstract

**Background:**

*Pseudomonas aeruginosa* is well known for its intrinsic ability to resist a wide range of antibiotics, thus complicates treatment. Thus, understanding the response of the pathogen to antibiotics is important for developing new therapies. In this study, proteomic response of *P. aeruginosa* to the commonly used anti-pseudomonas antibiotics, ceftazidime (Caz) and meropenem (Mem) was investigated.

**Methods:**

*P. aeruginosa* ATCC 9027, an antibiotic-susceptible strain, was exposed to sub-MIC values of antibiotics either Caz or Mem for 14 days to obtain E1 strains and then cultured in antibiotic-free environments for 10 days to obtain E2 strains. Proteomes of the initial and E1, E2 strains were identified and comparatively analyzed using isobaric tags for relative and absolute quantitation (iTRAQ) in cooperation with nano LC–MS/MS. Noted up and down-regulated proteins were confirmed with quantitative reverse transcriptase PCR (qRT-PCR).

**Results:**

Overall, 1039 and 1041 proteins were identified in Caz and Mem-exposed strains, respectively. Upon antibiotic exposure, there were 7–10% up-regulated (Caz: 71, Mem: 85) and down-regulated (Caz: 106, Mem: 69) proteins (1.5-fold change cut-off). For both Caz and Mem, the DEPs were primarily the ones involved in metabolic process, membrane, virulence, protein synthesis, and antibiotic resistance in which proteins involved in antibiotics resistance tended to be up-regulated while proteins involved in protein synthesis and metabolic process were down-regulated. Noted proteins included beta-lactamase AmpC which was up-regulated and OprD which was down-regulated in both the antibiotic-exposed strains. Besides, biofilm formation related proteins TssC1 and Hcp1 in Caz- exposed strains and the membrane/ periplasmic proteins Azu and PagL in Mem-exposed strains were found significantly down-regulated. qRT-PCR results confirmed the expression change of AmpC, Hcp1 and OprD proteins.

**Conclusion:**

Exposure of *Pseudomonas aeruginosa* to sub-MIC values of Caz and Mem resulted in around 10% change in its proteome. Not only proteins with confirmed roles in antibiotic resistance mechanisms changed their expression but also virulence- associated proteins*.* Both Caz and Mem response involved up-regulation of AmpC and down-regulation of OprD. While TssC1 and Hcp1 were responsible for Caz response, Azu and PagL were more likely involved in Mem response.

## Background

*Pseudomonas aeruginosa* (*P. aeruginosa*) is a well-known opportunistic Gram-negative bacterium that is known to cause infections in plants and animals [1]. In humans, this pathogen rarely affects healthy individuals. However, it has a significant morbidity and mortality rate in immunocompromised and cystic fibrosis (CF) patients, causing roughly 20% of acute and chronic infections [2]. Additionally, it is also responsible for approximately 10% of all nosocomial infections [2]. Due to the growing drug resistance, standard antibiotic regimens against *P. aeruginosa* are becoming increasingly ineffective [3].

Ceftazidime (Caz) and meropenem (Mem) are among standard anti-pseudomonas regimens. However resistant mechanisms of clinical *P. aeruginosa* are well recorded including altered membrane barrier, increased drug efflux pump, changed biofilm, resistant genes, and gene transfer [4–6]. Horizontal acquisition of beta-lactamases or altered expression of the chromosomal drug-inducible wide-spectrum class C beta-lactamase AmpC is the cause of a considerable proportion of Caz resistance [7]. MexAB-OprM, MexCD-OprJ, MexEF-OprN, and MexXY-OprM are the four most common Mex efflux systems and overexpression of MexAB-OprM efflux pumps could be the major source of Caz resistance in *P. aeruginosa* [8]. Regarding Mem resistance, the interaction between the efflux pump and porin D were important mechanisms [6]. Despite numerous investigations into the antibiotic resistance of *P. aeruginosa*, the mechanisms underlying the emergence of resistant traits, particularly in chronic infections remain unexplained [9].

Recently, the rapid development of novel proteomic technologies has allowed the detection of differential protein expression across samples in a single experiment. Isobaric tags for relative and absolute quantitation (iTRAQ) assay is an advanced high-throughput quantitative proteomics approach with excellent sensitivity that has been swiftly developed and widely utilized to explore the pathophysiology of a wide range of infectious pathogens and has been used to investigate a wide range of disorders, including depression, cancer, and cardiovascular disease [10, 11]. In this study, the response of *P. aeruginosa* to sub-MIC values of Caz and Mem was analyzed using comparative proteomic approach.

## Materials and methods

### Bacterial strains

*P. aeruginosa* ATCC 9027 (initial strain) was exposed to sub-MIC values of Caz and Mem by macro-dilution method in 24-well plate. The process was performed daily with the plate containing bacteria solution and the antibiotics diluted by the standard twofold dilution series in Muller-Hinton broth (MHB). The negative control (sterilized MHB) and the positive control (MHB and bacterial inoculum) were included in each plate. The plate was then incubated for 18 to 24 h at 37 °C. After that, the MIC value was recorded to evaluate the antibiotic resistance development of *P. aeruginosa* under the influence of Caz and Mem. Daily samples were collected and stored in 30% glycerol TSB at -80 °C. The 14^th^ day sample was designated as exposed-1 strain (Caz-E1, Mem-E1). The exposed-2 strain (Caz-E2, Mem-E2) was generated by cultivating the exposed-1 strain for 10 days in antibiotic-free media [12].

### Protein extraction and iTRAQ labeling

The bacteria protein was extracted by the sonication method and the concentration was measured by the Bradford kit. Briefly, cell pellets were collected from overnight broth culture by centrifugation at 13,000 rpm for 30 min at 4 °C. Cell membranes were disrupted by sonication and protein samples were collected after centrifugation. Protein samples were quantitated using a Bradford Kit (Bio-Rad, Hercules, CA) and checked the quality by SDS-PAGE electrophoresis, then stored at –80 °C for further analysis.

Protein samples were sent to be analyzed by the National University of Singapore. Briefly, iTRAQ labeling was performed using an iTRAQ Reagent Kit (AB SCIEX, Foster City, CA) according to the manufacturer’s protocol. Trypsin was added to lyse the protein overnight. The protein was labeled with the eight iTRAQ® Reagents – 8plex kit and incubated at room temperature. The iTRAQ-labelled mixture was desalted, purified, and analyzed by the liquid chromatography-tandem mass spectrometry (LC–MS/MS) and iTRAQ analysis.

### Proteomic analysis

Raw MS/MS data were analyzed using Protein Pilot Software 4.5 (AB SCIEX). Proteins were identified by searching the Swiss-Prot/UniProt protein database. For protein identification, a threshold applied was > 0.05 (CI, 10%) with setting ProtScore at 2.0 and FDR 1%. For analysis of differentially expressed proteins (DEPs), the proteins were considered as DEPs if their iTRAQ ratios were > 1.5 (upregulation) or < 0.667 (-1.5 after normalization: down-regulation) in exposed strains compared with the initial *P. aeruginosa* ATCC 9027.

A Venn diagram was constructed to analyze the common DEPs among exposed strains. Gene Ontology (GO) analysis software (PANTHER; Version 11.0, Protein Analysis Through Evolutionary Relationships; http://pantherdb.org) was used to evaluate the biological significance of the DEPs. Information on protein–protein interactions (PPIs) of the studied proteins was retrieved using the Search Tool for Retrieval of Interacting Genes/Proteins software (STRING; http://string-db.org/).

### Quantitative RT-qPCR

Noted DEPs identified via iTRAQ, were verified using RT-qPCR [13]. Briefly, total RNA was isolated by RNA isolation kit (New England Biolab, UK), according to the manufacturer’s protocol, cDNA was produced using the SensiFast cDNA synthesis kit (Meridan/Bioline, Canada). Primers (PHUSA Co, Vietnam) used in the study were listed in Table 1. Primers were designed using primerBLAST, checked with Primer3 and practically verified using gradient PCR. qRT-PCR was carried out with SensiFast SYBR no-ROX (Meridan/Bioline, Canada) according to the manufacturer’s protocol. Transcription values (Ct, cycle threshold) were analyzed as described in [14]. All experiments were done in triplicate. Foldchange and confidence level 95% CI (error bar) were calculated in MS Excel (Office 365, Microsoft Corporation) according to standard practice [15].

**Table 1:** Primers used in qRT-PCR

Genes	Sequence (5’-3’) (F: forward; R: reverse)	References
*rpsL*	F- CGGCACTGCGTAAGGTATGC R- CGTACTTCGAACGACCCTGCT	[16]
*rpoD*	F- GGGCGAAGAAGGAAATGGTC R- CAGGTGGCGTAGGTGGAGAA	[17]
*ampC*	F—CGGCGACATCAGCAACACC R—CGATGCTCGGGTTGGAATAGAGGC	[18]
*mexA*	F- GGCGACAACGCGGCGAAGG R- CCTTCTGCTTGACGCCTTCCTGC	[16]
*oprD*	F- GGGCCGTTGAAGTCGGAGTA R- GGCGACAACGCGGCGAAGG	[18]
*hcp1*	F-GACGTCAAGGGTGAGTCCAAGG R-CAGGTTGGGCGTGGACTTGTC	[19]

## Results

### Comparative proteome analysis of* P. aeruginosa*

**Fig. 1:**
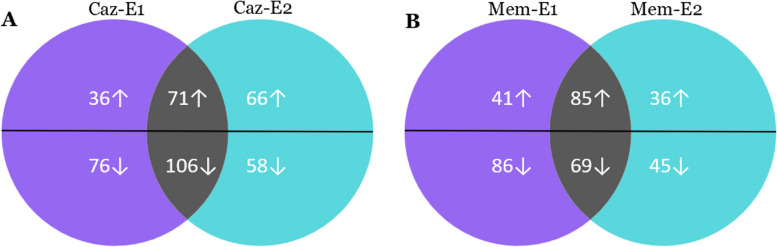
Comparison of DEPs found in Caz and Mem-exposed strains. **A** Total DEPs of Caz-exposed strains, **B** Total DEPs of Mem-exposed strains. Numbers of common DEPs were indicated at the intersections of the circles of the Venn diagram

In total, 1039 and 1041 proteins were identified in Caz and Mem-exposed strains, respectively. There were more down-regulated than up-regulated proteins (cut-off, 1.5-fold change). In Caz-exposed strains, Caz-E1 had 288 DEPs and Caz-E2 had 301 DEPs (see Fig. 1A). In addition, total 71 proteins were irreversibly up-regulated, and 106 proteins were irreversibly down-regulated after exposure to Caz. A total 281 DEPs were identified in Mem-E1 strain, including 155 that were down-regulated and 126 that were up-regulated. In Mem-E2 strain, there were only 235 DEPs, including 121 proteins that were up-regulated and 114 proteins were down-regulated (see Fig. 1B).

**Fig. 2:**
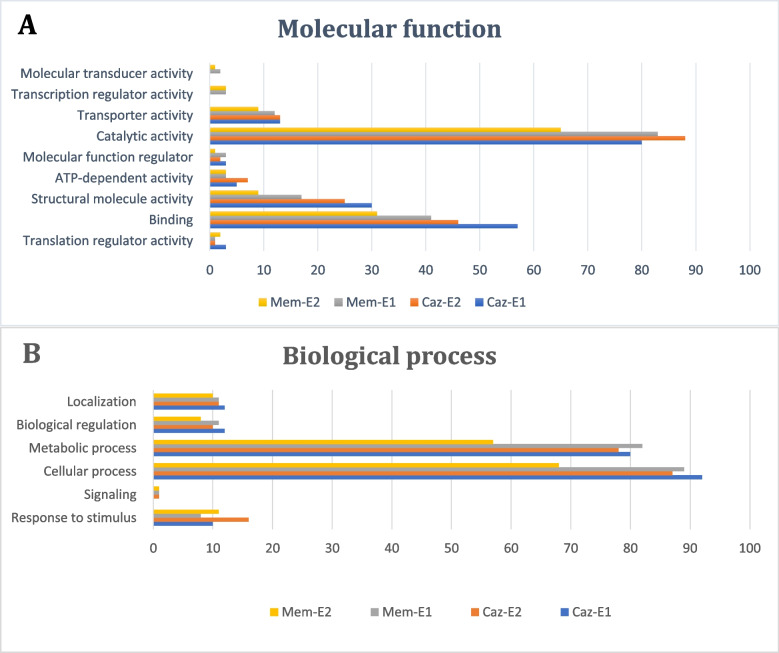
Classification of DEPs in Mem and Caz-exposed strains by (**A**). GO-Slim molecular function and (**B**). GO-Slim biological process. Annotation of DEPs was from PANTHER database

GO annotation of the DEPs of Caz- and Mem-exposed strains was classified into several categories and analyzed using Panther software. Similar DEP profiles were observed in both Caz- and Mem-exposed strains. Markedly changed DEPs involved in molecular functions, mostly in catalytic activity, binding, and structural molecule activity (Fig. 2A). The highest difference was found in proteins with catalytic activity ( Caz-E1: 80, Caz-E2: 88, Mem-E1: 83, Mem-E2: 65). However, only in Mem-exposed strains, DEPs of transcription regulators and molecular transducers were found. In addition, the Caz-E1 strain also did not have DEPs that are involved in signaling (Fig. 2B). The biological process annotation revealed that found DEPs were involved in four classes of GO terms, including cellular process and metabolic process, which were chemical reactions and pathways including anabolism and catabolism, small molecules transformation, and DNA repair and replication (Fig. 2).

#### Protein–protein interaction network* among DEPs*

**Fig. 3:**
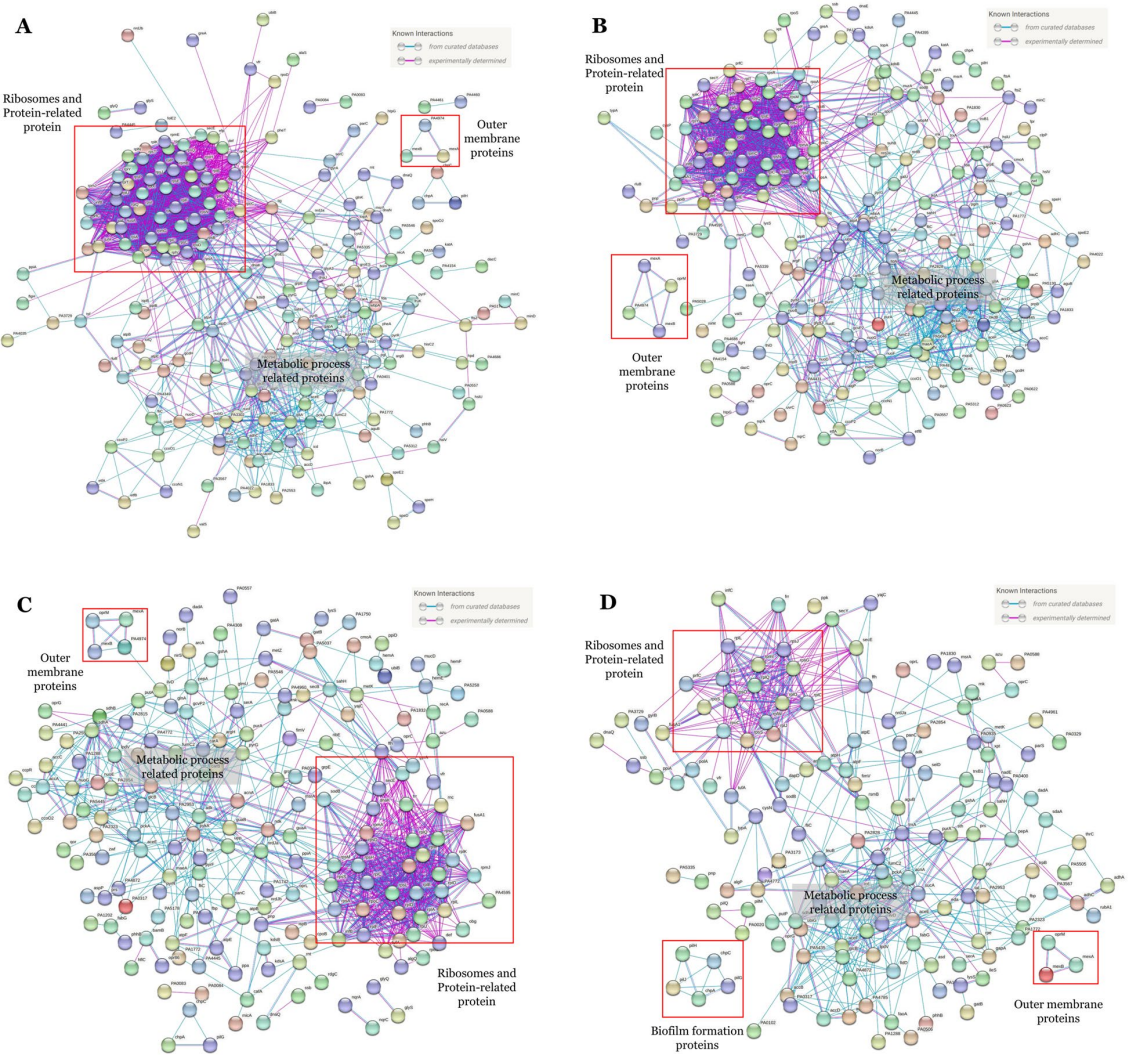
Protein–protein interaction network for DEPs of (**A**). Caz-E1, (**B**). Caz-E2, (**C**). Mem-E1, and (**D**). Mem-E2. Only known interactions were included. Disconnected nodes were hidden. Information on protein–protein interaction was retrieved using the STRING database

DEPs were classified using GO and PPI analysis for 290 DEPs of Caz-E1 (Fig. 3A), 308 DEPs of Caz-E2 (Fig. 3B), 281 DEPs of Mem-E1 (Fig. 3C), and 237 DEPs of Mem-E2 (Fig. 3D).

DEPs were mainly distributed in the ribosome, metabolism, DNA replication, regulation, and transmembrane transporter. These processes were all associated with transmembrane transport and protein biosynthesis, indicating the importance of these processes in Caz and Mem resistance in particular and antibiotic resistance in general.

#### Antibiotic resistance- related DEPs in Caz-exposed and Mem-exposed strains

 In both Caz-exposed and Mem-exposed, the proteomic analysis indicated that there were DEPs directedly involved in antibiotic resistance.

Notable DEPs that were associated with antibiotic resistance in Caz and Mem-exposed strains were shown in Tables 2 and 3, respectively. They included beta-lactamase AmpC, multidrug resistance proteins (MexA: Caz-E1_5.15, Mem-E1_9.73; MexB: Caz-E1_1.94, Mem-E1_2.54), which were upregulated and porin D (OprD: Caz-E1_-6.54, Mem-E1: -6.98) which was down-regulated in the both antibiotic exposed strains. In addition, down-regulated proteins were Azu (Caz-E1:1.02, Mem-E1: -4.70) and PagL (Caz-E1: -1.15, Mem-E1: -4.17) in Mem-exposed strains and TssC1 (Caz-E1: -3.87, Mem-E1: 1.54) and Hcp1 (Caz-E1: -18.18, Mem-E1: -1.79) in Caz-exposed strains.

**Table 2:** Some notable DEPs involved in antibiotic resistance in Caz-exposed strains

Accession #	Name	Caz-E1	Caz-E2
AMPC_PSEAE	Beta-lactamase	20.32	12.47
MEXA_PSEAE	Multidrug resistance protein MexA	5.15	4.74
MEXB_PSEAE	Multidrug resistance protein MexB	2.54	1.57
Q9I0Y8_PSEAE	Efflux pump membrane transporter	1.61	1.56
Q9HXU8_PSEAE	Lipotoxon F	2.01	2.31
RECA_PSEAE	Protein RecA	-1.71	-4.88
PORD_PSEAE	Porin D	-6.54	-3.13
Q9HWW1_PSEAE	Outer membrane protein OprG	-5.70	-1.79
TSSC1_PSEAE	Type VI secretion system sheath protein TssC1	-3.87	-3.34
HCP1_PSEAE	Protein Hcp1	-18.18	-8.87

**Table 3:** Some notable DEPs involved in antibiotic resistance in Mem-exposed strains

Accession #	Name	Mem-E1	Mem-E2
AMPC_PSEAE	Beta-lactamase	5.70	-5.34
MEXA_PSEAE	Multidrug resistance protein MexA	2.54	4.21
MEXB_PSEAE	Multidrug resistance protein MexB	1.94	1.91
OPRM_PSEAE	Outer membrane protein OprM	3.80	2.51
Q9HXU8_PSEAE	Lipotoxon F	5.11	4.41
Q9HZM1_PSEAE	Probable tolQ-type transport protein	8.17	-1.07
PORD_PSEAE	Porin D	-6.54	-3.13
RECA_PSEAE	Protein RecA	-2.05	-1.16
AZUR_PSEAE	Azurin	-4.70	-1.51
PAGL_PSEAE	Lipid A deacylase PagL	-4.17	-1.22

#### RT-qPCR reflected proteomic changes

**Fig. 4:**
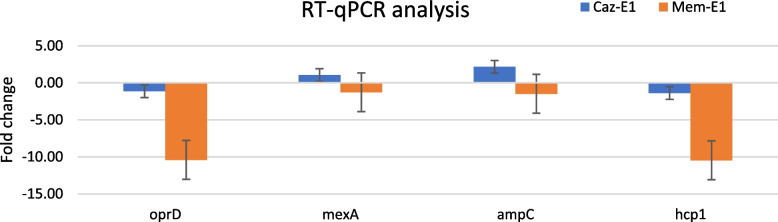
RT-qPCR analysis of selected genes in Caz-E1 and Mem-E1 strains. Fold change and confidence level 95% CI (error bar) were calculated using MS Excel. The average expression of housekeeping genes rspL and rpoD were used as the reference gene value [20]. Foldchange and confidence level 95% CI (error bar) were calculated in MS Excel according to standard practice [15]

Four genes (*oprD*, *mexA*, *ampC*, and *hcp1*) from Caz-E1 and Mem-E1 were selected for qRT-PCR analysis to quantify their transcriptional levels. Compared to the initial strain, there were decreases in *oprD* and *hcp1* gene expression in Caz-E1 and Mem-E1 (see Table 4, Fig. 4). On the other hand, *ampC* and *mexA* results showed a slight increase in Caz-E1 but a decrease in Mem-E1 (see Table 4, Fig. 4). The expression level trend of *oprD, mexA, ampC*, and *hcp1* was consistent in Caz-E1 between qRT-PCR and proteomics results. In Mem-E1, expression level trend was in agreement between qRT-PCR and proteomics results for *oprD* and *hcp1* but not for *mexA* and *ampC *(Table 4, Fig. 4). These results suggested that protein abundance was also affected by post- transcriptional and translational modifications.

**Table 4:** Comparison of normalized protein and mRNA fold change of Caz-E1 and Mem-E1 strains. Protein fold change was analyzed based on Itraq data. mRNA fold change was analyzed based on Ct values in RT-qPCR

Protein	Protein fold change		mRNA fold change	
	Caz-E1	Mem-E1	Caz-E1	Mem-E1
OprD	-6.54	-6.98	-1.14 ± 1.30	-7.29 ± 0.72
MexA	5.15	9.73	1.05 ± 1.54	-2.25 ± 0.35
AmpC	20.32	5.7	2.16 ± 5.48	-1.20 ± 1.49
Hcp1	-18.18	-1.79	-1.39 ± 0.03	-10.47 ± 2.17

## Discussions

*Pseudomonas aeruginosa* was a major opportunistic pathogen, causing a wide range of acute and chronic infections. Penicillins, carbapenems, monobactams, and cephalosporins were examples of beta-lactam antibiotics that are important in the management of *P. aeruginosa* infections. Noticeably, many *P. aeruginosa* isolates were resistant to beta-lactams, which complicated the management of infections and worsened patient outcomes [21]. Our study was designed to use a proteomic approach to highlight the physiological responses of *P. aeruginosa* regarding Caz-resistant and Mem-resistance mechanisms. In contrast to conventional biochemical procedures that only analyzed one or a few specific proteins, iTRAQ analysis paired with LC–MS/MS was a non-targeted research strategy for gene expression and might track the expression of numerous genes directly at the protein level [12]. This approach could offer comprehensive insights into how global proteins express differently under various physiological. Concomitant analysis of proteomic results from the Caz and Mem-exposed strains of *P. aeruginosa* facilitated our understanding of how this pathogen responds to Caz and Mem antibiotics.

**Fig. 5:**
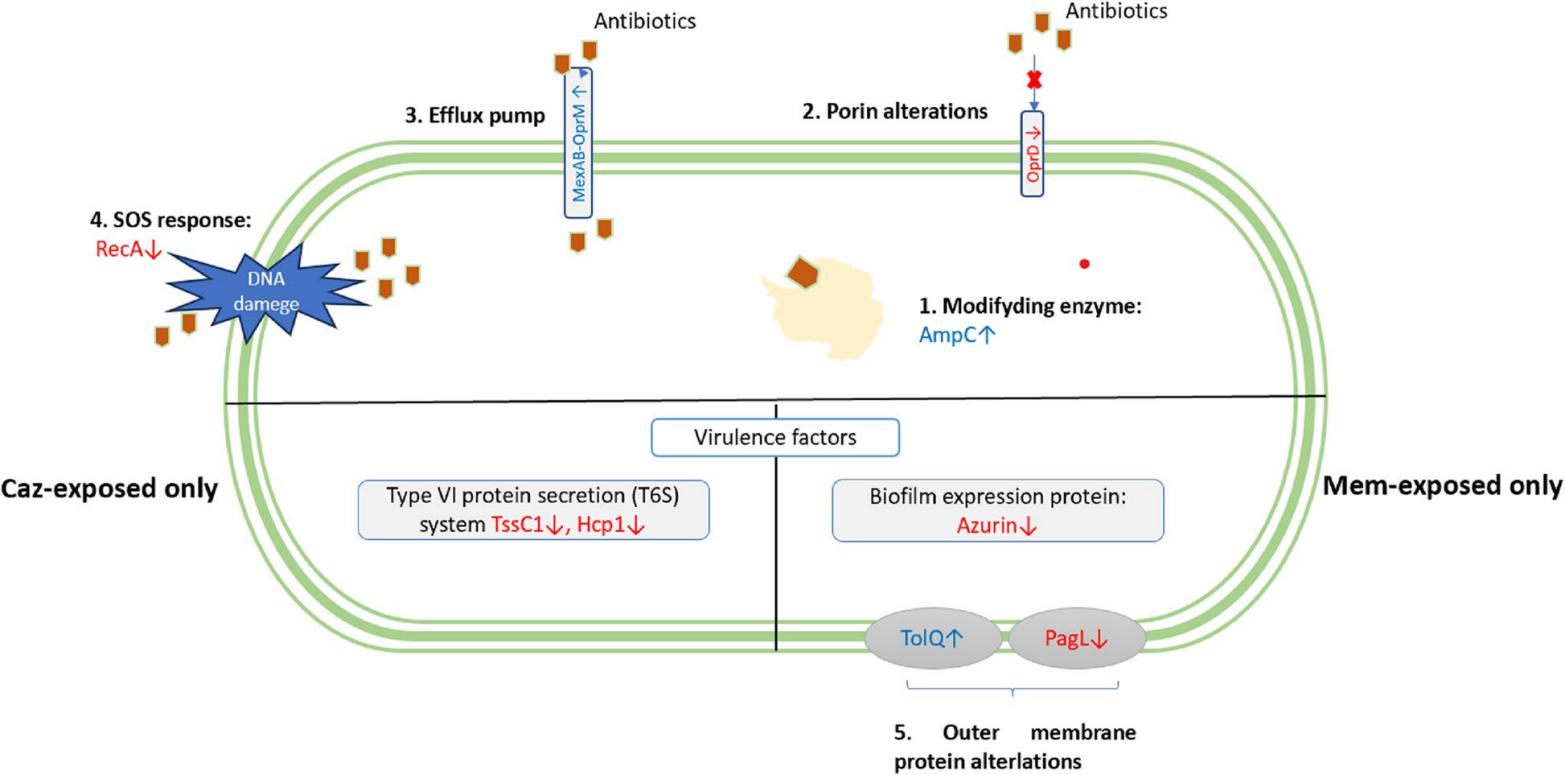
Proposed mechanisms for the development of Caz and Mem resistance. The common resistance mechanisms in Caz and Mem response: 1. Up-regulation of beta-lactamase AmpC, 2) Down-regulation of Porin D (OprD), 3) Up-regulation of efflux pump system (MexAB-OprM) and 4) Down-regulation of SOS response (RecA). The distinguished mechanisms: Type VI secretion proteins (TssC1 and Hcp1) showed their important role in Caz resistance, while Azurin (Azu) was important response in Mem resistance

The DEPs analysis showed a broad diversity of cellular functions which was affected the antibiotic exposure, including mainly the “metabolic process” and “cellular process”. One of notable DEPs is AmpC is found in both Caz and Mem response indicated its important role. In clinical bacterial isolate, the *ampC* mutation led to AmpC overproduction [22, 23]. In our study, the regulator for AmpC, including AmpR, AmpD, and AmpG was not identified among all strains. AmpC beta-lactamase was able to hydrolyze cephalosporins despite the low concentration of the substrate [24]. Furthermore, reduced number of porin entry channels or increased production of efflux pumps could reduce the antibiotic inflow and further enhance enzyme efficiency. Interestingly, in this study, AmpC upregulated significantly in Mem-E1 (5.70) when Mem presented in the culture then reduced markedly in Mem-E2 (-5.34) when Mem was no longer in the culture. In contrast, in Caz response, AmpC was highly upregulated even when Caz was removed (Caz-E1: 20.32, Caz-E2: 12.47) (Fig. 5).

As mentioned earlier, membraned proteins included efflux pumps and porin have high impact on antibiotic resistance [25, 26]. In this study, OprD showed significant down-regulation in Caz (Caz-E1: -6.54) and Mem-exposed (Mem-E1: -6.98) strains which well aligned with previous findings [23].

The Mex efflux pump system, MexAB, which were expressed together with OprM to form one of the most important efflux pumps of *P. aeruginosa* resulted in the ability of *P. aeruginosa* to resist multiple antibiotics and its overexpression confers cross-resistance or reduced susceptibility to several antibiotics [27]. In the present study, the MexAB-OprM was also found increased in Caz response in *Pseudomonas aeruginosa*.

LptF, outer membrane protein that involves in the adhesion to lung epithelia and resistance to reactive oxygen species [28]. The up-regulation of LptF (Caz-E1: 2.01, Mem-E1: 5.11) contributed to the increase of virulence in the presence of beta-lactam antibiotics. Besides, the role of Tol-pal system (TolQ) and PhoP/PhoQ-induced Lipase/ Lipid A deacylase (PagL) is noticeble. Mem-E1 showed the up-regulation in TolQ (Mem-E1: 8.17) and down-regulation in PagL (Mem-E1: -4.17, Mem-E2). TolQ and PagL were known to be involved in antibiotic resistance because of their ability in maintenance of outer membrane integrity in Gram-negative bacteria [29–31] (Fig. 5).

Two significantly decreased DEPs in Caz and Mem response were Hcp1 and TssC1 proteins which were components of the type VI (H1-T6SS) secretion system. These proteins involved in biofilm formation and virulence of *P. aeruginosa* [32]. These proteins were observed to be significantly decreased in Caz-exposed and Mem-exposed strains. The clear decrease of Azu in Mem- exposed strains was seen not only in this study but also in a previous study working with polymyxin resistance [33] (Fig. 5). The role of this small electron donor protein in antibiotic resistance still remains unclear and should be further investigated.

## Conclusions

In conclusion, most DEPs associated in antibiotic response were associated with stress responses, cellular components, metabolism, protein synthesis, and virulence. Common proteomic response to Caz and Mem involved AmpC and OprD*.* While Azu and PagL were more likely involved in Mem response, TssC1 and Hcp1 were responsible for Caz response.

## Data Availability

The datasets used and/or analysed during the current study are available from the corresponding author on reasonable request.

## Abbreviations

*P. aeruginosa*: *Pseudomonas aeruginosa*
ATCC: American type culture collection
iTRAQ: Isobaric tags for relative and absolute quantitation
LC–MS/MS: Liquid chromatography with tandem mass spectrometry
Caz: Ceftazidime
Mem: Meropenem
MHB: Mueller hilton broth
MHA: Mueller hilton agar
DEP: Differentially expressed protein
CF: Cystic fibrosis
PPI: Protein–protein interaction
qRT-PCR: Quantitative reverse transcriptase PCR
GO: Gene ontology
